# Will wealth inequality decrease happiness?—Empirical evidence from China

**DOI:** 10.3389/fpsyg.2023.1259456

**Published:** 2024-02-01

**Authors:** Jingtao Wang

**Affiliations:** School of Advanced International Studies, Johns Hopkins University, Washington, DC, United States

**Keywords:** wealth inequality, happiness, relative deprivation, Kakwani index, China

## Abstract

**Introduction:**

This article delves into the broad social and economic impacts of wealth inequality, specifically focusing on its effects on happiness, as analyzed using micro survey data from the China Family Panel Studies (CFPS).

**Methods:**

This article employs the panel OLS regression method with time and province fixed effects for the main result and heterogeneity analysis, then uses the mediating effect and moderating effect test for the mechanisms.

**Results and discussion:**

The article presents several key findings: 1. Impact of Wealth Inequality on Happiness. The study confirms that wealth inequality significantly reduces happiness, a conclusion reinforced by a range of consistency tests and endogeneity checks. 2. Heterogeneity Analysis. Three areas of heterogeneity are examined: Hukou status, education level, and family members’ average income. The results indicate that the happiness of families with a family head holding an urban Hukou, higher education, or a higher per-member income level is less affected by wealth inequality. 3. Mechanisms Affecting Happiness. At the micro-level, the article identifies two mediating pathways—health and marital status—through which wealth inequality negatively influences happiness. At the macro-level, it is found that social security expenditure and economic development can moderate these effects and enhance subjective happiness under the same conditions of wealth inequality. The contributions of this study are specific as: 1. This study addresses some of the existing gaps in the research regarding the relationship between wealth inequality and happiness. 2. The article utilizes relative deprivation as a measure of wealth inequality, considered a more apt metric for studying happiness compared to absolute inequality. 3. This research offers insights into the mechanisms behind the observed effects, considering both micro-level (individual and family) and macro-level (societal and economic) factors.

## Introduction

In today’s increasingly diverse world, the paradigm of human flourishing extends beyond mere economic development. The pursuit of happiness, once a secondary consideration, has now become a central focus in governmental policymaking, as noted by Ura et al. (2022). This shift was marked by the United Nations’ inaugural happiness report in 2012, which prompted various countries to integrate the pursuit of happiness into their national objectives. Bhutan emerged as a forerunner in this movement, emphasizing citizen happiness. Following suit, developed nations such as the United Arab Emirates, New Zealand, Finland, Scotland, Japan, and others have also begun to prioritize the happiness of their citizens in their national agendas. China, notable for its rapid economic growth in recent decades, has similarly adopted happiness as a key indicator of societal progress. However, societal and economic challenges, particularly wealth inequality, have emerged as significant obstacles to the advancement of citizen happiness, necessitating urgent attention and solutions.

### Wealth inequality

While wealth inequality has a long-standing history, it has only recently become a subject of focused academic inquiry, largely due to improvements in data availability. Contemporary literature offers insights into the current state of wealth inequality, both globally and within China. Zucman (2019) examined changes in wealth inequality across the United States, the United Kingdom, France, China, and Russia. His findings revealed a significant positive correlation between higher rates of wealth growth and the increased wealth of the top 1% of households. Zucman also underscores the challenge of accurately measuring wealth inequality due to the prevalence of offshore wealth. In a related study, Alstadsæter et al. (2018) discovered that approximately 10% of the world’s GDP is held in offshore tax havens, with considerable variation across regions. For example, the proportion of wealth stored in tax havens is relatively low in Scandinavian countries, but it rises to about 15% in continental Europe and even higher, up to 60%, in certain Latin American and Gulf countries.

Recent data on China’s wealth reveal rapid growth accompanied by distinct trends in wealth allocation and asset preferences. According to the Boston Consulting Group’s (BCG) 2020 Global Wealth Report, a significant 69% of China’s wealth is invested in real assets, a stark contrast to the 24% in the United States and 55% in Western Europe. The China Statistical Yearbook, issued by the National Bureau of Statistics of China, indicates a growing trend toward diversification in the allocation of China’s financial assets. Chinese investors are increasingly moving away from traditional savings accounts, favoring market investments instead. This shift is evidenced by a pronounced negative correlation between investments in real estate and financial assets, with individuals often transferring funds between markets based on performance. These specific trends in China’s investment landscape have been contributing factors to the escalating wealth inequality within the country. Data from the World Wealth and Income Database reveal that the wealth growth rate for China’s top 10% far exceeds that of the remaining 90%. In 2014, this affluent top 10% held over 60% of the nation’s wealth, up from less than 50% in the early 2000s. Further emphasizing this disparity, 26% of China’s financial assets are in the hands of high- and ultra-high-net-worth individuals, with affluent individuals collectively holding 40% of these assets, leaving the majority population with the remaining 60%.

Piketty et al. (2019) employed the generalized Pareto interpolation method to assess wealth inequality in China. Their findings revealed a stark increase in the wealth share of the top 10%, which grew from 40% in 1995 to 67% in 2015. Conversely, the wealth share of the middle 40% declined from 43 to 26%, and the bottom 50% of the population held less than 7% of total wealth by 2015. In a separate analysis, Wan and Knight (2023) examined wealth inequality trends between 2002–2013 and 2013–2018. They noted a deceleration in the growth rate of wealth inequality, though it remained a significant challenge, partly due to inadequate policy responses from the Chinese government. Adding to this perspective, Credit Suisse’s 2021 Global Wealth Report indicated a fluctuating trend in China’s Gini coefficient, a measure of inequality: it rose from 0.599 in 2000 to 0.711 in 2015, decreased slightly to 0.697 in 2019, but then surged to 0.704 in 2020 amidst the COVID-19 pandemic. These data, though derived from a different source, corroborate the ongoing challenge of addressing wealth inequality in China.

In addition to acknowledging the current state of wealth inequality globally and in China, extensive research has been conducted on its economic impacts. At a macroeconomic level, Piketty (2014) notes that wealth inequality undermines economic efficiency and exacerbates income and social disparities. This leads to reduced economic growth and results in varying steady states, as described by Galor and Zeira (1993). Furthermore, wealth inequality increases the dependency of middle- and low-value households on leverage, while high-value households seek greater capital returns, promoting the growth of high-leverage investment vehicles. These factors collectively contribute to economic instability (Kumhof et al., 2015; Goda et al., 2017). On a microeconomic scale, wealth inequality hampers the accumulation of human capital in lower-value families, a trend that persists across generations (Bhattacharya et al., 2016; Lusardi et al., 2017). Moreover, Chikhale (2023) found that in environments of high wealth inequality, consumption growth is more adversely affected by uncertainty shocks. Beyond economic factors, wealth inequality also has varying impacts on physical and mental health (Ostendorf et al., 2001; Lorgelly and Lindley, 2008). It further diminishes the inclination of individuals with low net wealth to engage in entrepreneurship (Gentry and Hubbard, 2000) and increases their propensity to abandon such ventures (Frid et al., 2016).

### Wealth–happiness relationships

The exploration of the link between wealth inequality and happiness, as highlighted in Okulicz-Kozaryn (2022), is a relatively nascent area of study. It expands on the well-known “Easterlin Paradox” (Easterlin, 1974), which delves into the puzzling relationship between income and happiness. This paradox emerges from the conflicting outcomes observed in cross-sectional and time-series data. Cross-sectional data suggest that within a given country, individuals with higher incomes tend to report greater happiness. In contrast, time-series data from the same country often lead to a different conclusion, indicating that increases in income do not necessarily translate to enhanced happiness. Various theories have been proposed to reconcile this paradox. One notable theory is the “relative income hypothesis” (Easterlin, 2003), which posits that once basic life necessities such as food, clothing, and housing are met, happiness becomes less dependent on absolute income and more influenced by income relative to one’s peer group. Another proposed explanation is the “set point theory,” which argues that happiness levels tend to stabilize after basic living standards are achieved. According to this theory, happiness can be temporarily affected by positive or negative life events but ultimately returns to a baseline state over time (Brickman et al., 1978).

Wealth is often perceived as an accumulation of income, serving as a means to transfer consumption ability from the past into the future, as described by Clark et al. (2008). It plays multiple roles in both directly and indirectly influencing happiness. Hauser (2007) outlines seven distinct functions of wealth that contribute to increased happiness: the security function—providing a sense of financial safety; the income function—its ability to generate income; the use function—its utility in consumption; the power function—enhancing one’s influence; the social status attainment and positioning function—elevating an individual to a “happier” status; and the inheritance function—allowing wealth to be passed down through generations. This perspective suggests that wealth may be an overlooked factor in prior income–happiness studies, as low income does not necessarily equate to low wealth, and wealth impacts happiness in more ways than income alone. Supporting this view, Kasinger et al. (2023) found that net wealth is a significant predictor of life satisfaction, based on data from the German Socio-Economic Panel (GSOEP). Nonetheless, research in the area of wealth and happiness remains relatively limited.

In research focused on China, Li et al. (2015) were among the first to investigate the relationship between assets, debts, and happiness. Using data from the China Household Finance Survey (CHFS), they examined how various assets and debts impact individual happiness levels. Subsequently, Wang and Shi (2022) explored how wealth inequality affects fertility intentions among Chinese households, discovering differing impacts of asset ownership between urban and rural areas. Ge et al. (2021) delved into the influence of opportunity inequality on wealth inequality, applying Roemer’s “environment effort” dualistic analysis framework. Their analysis, also based on CHFS data, revealed a significant positive correlation between opportunity inequality and wealth inequality, leading to a self-perpetuating cycle of wealth disparity. Wu et al. (2017) studied the interplay between wealth inequality and financial literacy in household borrowing. They found that increased financial literacy boosts both the likelihood and extent of household debt, with varying effects between rural and urban populations. Additionally, their research indicated that wealth inequality decreases the overall demand for debt and mitigates the impact of financial literacy on borrowing behaviors.

Upon reviewing the literature, three studies with a similar focus have been identified. The first, Wang et al. (2019), investigated the relationship between happiness and both absolute and relative wealth, the latter measured using a ranked ratio. Their findings suggest that wealthier individuals tend to be happier and that happiness correlates positively with higher wealth rankings. However, this study presents a few potential limitations. Firstly, despite the use of panel data, the individuals observed were not consistently present throughout all periods; their participation was intermittent. Secondly, the method employed to measure relative wealth inequality might lack precision. The ranking system used does not effectively capture the exact differences in wealth, which could influence the study’s accuracy in assessing the relationship between wealth and happiness.

The second noteworthy study is by Kasinger et al. (2023), which determined that net wealth is a highly significant predictor of life satisfaction, based on analysis of the German Socio-Economic Panel (GSOEP) data. This research centers on the impact of absolute wealth on life satisfaction, including an examination of the divergent wealth trajectories in West and East Germany. However, the focus of this study is on objective wealth rather than on subjective comparisons of wealth. According to the relative income hypothesis, subjective perceptions of wealth, rather than its objective value, are likely to have a more substantial influence on happiness. This aspect of wealth’s influence on happiness, rooted in subjective comparison rather than objective measurement, is not addressed in Kasinger et al.’s study.

The third study of interest is by Gao et al. (2022), which employs the Gini coefficient as a measure to interpret wealth inequality. Their research revealed a negative correlation between wealth inequality and subjective happiness, elucidating this relationship through the lens of life cycle theory. However, as acknowledged in their article, the data used are cross-sectional rather than panel in nature. This type of data collection is less optimal for comparing changes in happiness over different years, as it does not track the same individuals over time. The insights gained from these literature reviews lead to the formulation of our hypothesis.

### Hypothesis: there is a negative linear association between wealth inequality and happiness

Although research in this field is limited, we posit that certain mechanisms driving the expansion of wealth inequality may impact people’s behavior and subjective satisfaction. Our hypothesis draws on literature such as Piketty’s (2014) discussion of the contentious r-g theory, where the rate of return on capital (r) outpaces the growth rate of income (g). This disparity suggests that wealth inequality may persist without convergence. Complementing this, Benhabib et al. (2017) examined the relationship between earnings distribution and wealth distribution, concluding that income alone cannot fully explain the significant disparity in wealth distribution; factors such as savings rate and return on wealth also play critical roles. Both studies underscore the challenge in mitigating wealth inequality, which, in turn, could diminish happiness. Additionally, factors such as rising real estate prices in China (Li and Wan, 2015; Wan et al., 2021) exacerbate wealth inequality. Moreover, health issues (Omer et al., 2014) and challenges in marriage (Coontz and Folbre, 2002), often developed from wealth inequality, can alter people’s behaviors and potentially harm their happiness.

If our hypothesis is disproven, there are studies that could potentially support this refutation. Bagchi and Svejnar (2015) argued that wealth inequality, when not intertwined with political factors, does not negatively impact a country’s economic growth. Frid et al. (2016) demonstrated that wealth inequality does not necessarily deter individuals from pursuing entrepreneurship. Additionally, Bardhan et al. (2007) suggested that a certain level of wealth inequality may actually facilitate optimal collective action. These findings indicate that wealth inequality may have a neutral or even positive effect on economic growth and on behaviors at both the individual and group levels.

In this research, we delve into the impact of wealth inequality on happiness. Our goal is not only to assess the relationship between these two factors but also to explore the underlying mechanisms that may explain this relationship. This article aims to shed some light on the research on wealth inequality and happiness. While existing literature has extensively examined income inequality and its relation to happiness, there remains a significant gap in understanding the specific impact of relative wealth inequality on happiness. Our article distinguishes itself as the first to rigorously evaluate the effect of relative wealth inequality on happiness using panel data. This study introduces three innovative approaches to enrich the field: 1. It employs the Kakwani Index to measure wealth inequality, considering both the ranking of inequality and the actual differences in wealth between households; 2. It utilizes panel data, enabling an examination of the effects on households over two different time periods; 3. It identifies potential influencing mechanisms at both the micro level (household) and the macro level (policy).

## Data and methodology

### Data description

In our study, we have utilized data from the China Family Panel Studies (CFPS), conducted by the Institute of Social Science Survey (ISSS) at Peking University. This survey, initiated in 2010, is carried out biennially across 34 provinces in China. Given that CFPS provides panel data, we have chosen to analyze the data from the same families collected in both 2012 and 2018. After filtering out unmatched families, our final sample comprised observations from 6,782 families for both these years. The CFPS dataset includes a comprehensive range of economic and non-economic variables and measures of happiness, thereby offering a robust foundation for testing our hypotheses. We have collated the definitions of all the variables used in our analysis in Table 1.

**TABLE 1:** Variables definition.

Main variables	Definition
happiness	Very happy, Happy, Generally, Unhappy, and Very unhappy (with values from 5 to 1).
wineq	Kakwani index representation of relative deprivation of wealth inequality based on nominal net wealth.
**Control variables**	
male	The family head is male: the value of 1; the family head is female: the value of 0.
age_60	The family head age is greater than 60: the value of 1; otherwise: the value of 0.
edu_year	The years of schooling of the family head.
religion	The family head has a religion: the value of 1; otherwise: the value of 0.
familysize	The number of family members with financial dependence.
youth_rate	The number of family members with ages less than 14.
elder_rate	The number of family members with ages greater than 60.
lfamily_income_n	Log value of average nominal family income.
house	The family owns a house: the value of 1; otherwise: the value of 0.
med	The family has encountered catastrophic health payments, defined by the total health payment of the year greater than 40% of total family income.
**Tests of consistency variables**	
happiness_d	Dummy variable of happiness: the happiness score is greater than 3, the value of 1; otherwise, the value of 0.
wineq_adj	Kakwani index representation of relative deprivation of wealth inequality based on real net wealth.
lfamily_income_r	Log value of average real family income.
**Heterogeneity analysis variable**	
hukou	The family head obtains a rural hukou: the value of 0; the family head obtains an urban hukou: the value of 1.
**Mechanism test variables**	
health	Very unhealthy, Unhealthy, Generally, Healthy, Very healthy (with values from 5 to 1).
marriage	The family head is married: the value of 1; otherwise: the value of 0.
social_security_exp	Percentage of provincial social security expenditure against total government expenditure.
wineq × social_security_exp	Interactive variable between Kakwani index and social security expenditure percentage.
ngdppc	Log value of nominal provincial GDP per capita.
wineq × ngdppc	Interactive variable between Kakwani index and log value of nominal provincial GDP per capita.

### Measures

#### Happiness

Firstly, it is essential to clarify our definition of happiness. Khalil (2019) differentiates well-being, described as a corporeal utility derived from physical inputs such as food and clothing, from happiness. Happiness is characterized as a transcendental utility that emerges from evaluating corporeal utility (content) against one’s perceived ideal well-being (context). Therefore, happiness represents the subjective experience at the moment when individuals attain objective utility. Expanding on this, Khalil (2022) discusses the dual aspects of happiness: the happiness of tranquility, where individuals compare their present state to their past, and the happiness of aspiration, involving comparisons between the present and a potential future. According to Khalil, the happiness of tranquility is typically reflected in cross-sectional data, while the happiness of aspiration is captured in time-series data. In our research, utilizing the CFPS data, which are panel in nature, we have the unique opportunity to concurrently address both aspects of happiness.

The CFPS dataset includes individual responses related to subjective well-being. For consistency in our analysis, we used responses to the question “Are you satisfied with your life?” as an indicator of an individual’s happiness. In both the 2012 and 2018 datasets, responses are categorized on a five-point scale ranging from (1) Very unsatisfied to (5) Very satisfied. Previous research, such as that of Fowler and Christakis (2008), suggests that the happiness of the family head can be indicative of the overall happiness of the family. In our sample, we identified the family head as the primary respondent in the family economic survey. This decision is based on two considerations: firstly, the CFPS dataset does not explicitly identify who the family head is, and secondly, given our focus on wealth inequality, the primary respondent of the economic survey is likely the most knowledgeable about the family’s wealth and possibly the main decision-maker on significant family matters. Our analysis of the panel data from both years revealed an average happiness score of 3.629, which falls between neutral and satisfied. Moreover, we observed an increase in happiness from 2012 to 2018. This finding contrasts with earlier studies, such as those by Brockmann et al. (2009) and Knight and Gunatilaka (2011), which reported a decline in happiness over later periods. The detailed responses regarding happiness for both years are compiled in Table 2.

**TABLE 2:** Detailed happiness score.

Happiness score	Frequency	Percent	Cumulative percent
1	601	4.43	4.43
2	1,119	8.25	12.68
3	4,586	33.81	46.49
4	3,658	26.97	73.46
5	3,600	26.54	100.00
Total	13,564	100.00	

#### Wealth inequality

In this article, our focus is on wealth inequality rather than income inequality, due to its more permanent nature. We aim to explore how changes in relative wealth inequality impact happiness, drawing on the principles of the social comparison phenomenon and relative deprivation theory.

To quantify wealth inequality, we have ranked families based on their net assets, using a winsorized (1,99) approach to mitigate the impact of extreme values. The Kakwani Index (KI) served as our measure of relative deprivation in wealth inequality (He et al., 2021). Developed as an improvement on the Yitzhaki Index, the KI addresses issues related to sensitivity to changes in sample scale (Adjaye-Gbewonyo and Kawachi, 2012). In our methodology, we calculated the KI based on the net assets of a family positioned at a specific rank (*k*) in both 2012 and 2018. This approach enabled us to assess the relative wealth position of each family over these two periods.


(1) $$RD\left(x,x_{k}\right)\;=\;\frac{1}{n{\text{μ}}_{X}}\sum_{i=k+1}^{n}\left(x_{i}-x_{k}\right)\text{ = }\gamma_{xk}^{+}\left[\left({\text{μ}}_{xk}^{+}-x_{k}\right)/{\text{μ}}_{X}\right]$$

*x_k_* represents the net asset for the family ranked at *k*, *x_i_* represents the net asset of a family ranked higher than *k*, *n* represents the total number of families, and μ_*X*_ represents the mean net asset of all families. The right-hand side formula is another version of KI, in which $\gamma_{x\,k}^{+}$ represents the percentage of the sample that has the higher net assets, and $\mu_{x\,k}^{+}$ represents the mean of all families’ net assets higher than the rank *k*family.

Upon computing wealth inequality using the Kakwani Index (KI), we discovered that the average KI stands at 0.637. This figure aligns closely with the average wealth Gini coefficient observed for the years 2012 and 2018. Notably, the lowest recorded KI value is 0, a common result for individuals at the highest end of the wealth spectrum, who typically do not experience feelings of deprivation. However, an intriguing finding in our analysis is the maximum KI value of 1.087, which deviates from the usual range of 0 to 1 for KI. This anomaly can be attributed to the unique nature of wealth calculation, where debts may exceed assets, resulting in negative net assets. Consequently, in such scenarios, the KI can exceed the typical upper limit of 1.

#### Control variables

In our analysis, we accounted for variables at both the individual and family levels that might influence happiness. On the individual level, our controls included the gender of the individual, age (specifically, being older than 60), total years of education, and religious affiliation (specifically, belief or not in a religion). At the family level, we considered factors such as family size, the proportion of young and elderly members within the family, the logarithm of average net income per family member, home ownership status, and the presence of catastrophic health payments. We believe these variables aptly reflect the demographic and economic circumstances of a family and its head. For ease of reference and clarity, we have compiled all these control variables in Table 3 under the “Control Variables Sector.” This comprehensive approach ensures a thorough consideration of the various factors that could potentially impact the happiness of individuals and families.

**TABLE 3:** Variables summary.

Main variables	Obs	Mean	Std. Dev.	Min	Max
happiness	13,564	3.629	1.093	1	5
wineq	13,564	0.637	0.245	0	1.087
**Control variables**					
male	13,564	0.512	0.5	0	1
age_60	13,564	0.278	0.448	0	1
edu_year	13,564	6.42	4.564	0	15
religion	13,564	0.077	0.266	0	1
familysize	13,564	3.896	1.792	1	15
youth_rate	13,564	0.084	0.14	0	0.75
elder_rate	13,564	0.196	0.294	0	1
lfamily_income_n	13,564	9.118	1.291	−9.210	14.421
house	13,564	0.9	0.3	0	1
med	13,564	0.114	0.317	0	1
**Tests of Consistency Variables**					
happiness_d	13,564	0.535	0.499	0	1
wineq_adj	13,564	0.634	0.246	0	1.087
lfamily_income r	13,564	8.751	1.966	−9.210	13.649
**Heterogeneity Analysis Variable**					
hukou	13,564	0.255	0.436	0	1
edu_year = 0	3,734	0	0	0	0
edu_year = 6	3,464	6	0	6	6
edu_year = 9	4,011	9	0	9	9
edu_year > = 12	2,355	12.838	1.346	12	15
family_income_r 1st quartile	3,392	1537.57	1031.37	0.0001	3490.00
family_income_r 2nd quartile	3,390	5973.60	1493.04	3497.33	8640.00
family_income_r 3rd quartile	3,391	12474.94	2466.64	8641.50	17175.00
family_income_r 4th quartile	3,391	35403.28	28773.00	17180.00	84666.66
**Mechanism Test Variables**					
health	13,564	3.283	1.214	1	5
marriage	13,564	0.879	0.326	0	1
social_security_exp	13,564	0.138	0.041	0.079	0.274
wineq × social_security _exp	13,564	0.01	0.031	−0.169	0.225
ngdppc	13,564	4.643	0.192	4.278	5.179
wineq × ngdppc	13,564	0.292	1.008	−4.779	4.814

### Model specification

Reflecting on the literature we reviewed, it appears that simply measuring absolute wealth differences might not fully capture the variations in happiness among individuals. Therefore, we propose to use relative deprivation in wealth as the key independent variable to explain these differences in happiness.

Thus, to test the causal relationship between relative wealth differences and happiness, we planned to employ a panel OLS regression model. While the ordered probit model is commonly used in happiness studies, Ferrer-i-Carbonell and Frijters (2004) have noted that OLS regression can yield results comparable to those of the ordered probit model in this context. This approach is also consistent with earlier research in this field, such as the works of Gao et al. (2022) and Kasinger et al. (2023), who used linear regression models in their analyses.

Given that our study is based on panel data, we have incorporated fixed effects for time to control for year-specific factors. Additionally, we considered the inclusion of both time and provincial fixed effects to account for variations across different years and locations.

First, we assumed that happiness is dependent on the relative wealth, year, and family-specific factors:


(2) $$h\,a\,p\,p\,i\,n\,e\,s\,s_{i\,t}=\beta_{0}+\beta_{1}\,R\,D_{i\,t}+X_{i\,t}+\mu_{i}+\lambda_{t}+\varepsilon_{i\,t}$$

The *happiness*_*it*_ is the observed happiness for family *i* at year *t*, *RD*_*it*_ is the wealth inequality (wineq) index of an individual assigned from family wealth at year *t* based on KI, *X*_*it*_ is the control variables for family *i* in year *t*, μ_*i*_ is the province fixed effect, λ_*t*_ is the time fixed effect, and ε_*it*_ is the error term for the individual family *i* in year *t*.

Upon deriving our main findings, we developed three different methods to test the consistency of our primary result. After the test of consistency, we analyzed the potential heterogeneity within the relationship between happiness and wealth inequality. The specific variables used are outlined in Table 3.

### Mechanism test models

Following our rigorous validation of the main results through various tests of consistency and analysis of heterogeneity, our next step was to propose and evaluate the potential mechanisms underlying these findings. Specifically, we aimed to identify and test the influential factors at both the micro and macro levels that could affect the magnitude of the relationship between wealth inequality and family happiness. This aspect of our analysis involved examining both the mediating and moderating effects of different variables, including both the individual and societal factors, to determine how they might intensify or mitigate the impact of wealth inequality on subjective happiness. The complete list of variables used for testing these mechanisms is meticulously compiled in Table 3 under the “Mechanism Test Variables” section.

#### Mediating effect model and variables

In our approach to assessing the mediating effect, our methodology involved a two-step regression analysis. Initially, we regressed wealth inequality along with other control variables against the chosen mediating variable. This step aimed to understand how wealth inequality and other relevant factors influence the mediating variable. Following this, the second regression was conducted, where the mediating variable, wealth inequality variable, and other control variables were regressed together against happiness. This step is crucial as it elucidates how the mediating variable interacts with wealth inequality to impact happiness. The mediating effect model is presented below:


(3) $$S\,t\,e\,p\,1:m\,e\,d\,i\,a\,t\,i\,n\,g_{i\,t}=\gamma_{0}+\gamma_{1}\,R\,D_{i\,t}+X_{i\,t}+\mu_{i}+\lambda_{t}+\varepsilon_{i\,t}$$

$$S\,t\,e\,p\,2:h\,a\,p\,p\,i\,n\,e\,s\,s_{i\,t}=\beta_{0}+\beta_{1}\,R\,D_{i\,t}+\beta_{2}\,m\,e\,d\,i\,a\,t\,i\,n\,g_{i\,t}+$$

(4) $$X_{i\,t}+\mu_{i}+\lambda_{t}+\varepsilon_{i\,t}$$

The *mediating*_*it*_ is the mediating effect variable for household *i* at year *t*. In our study, we considered health and marriage to be two mediating variables. The *happiness*_*it*_ is the observed happiness for family *i* at year *t*, *RD*_*it*_ is the wealth inequality (wineq) index of an individual assigned from family wealth at year *t* based on KI, *X*_*it*_ is the control variable for family *i* in year *t*, μ_*i*_ is the province fixed effect, λ_*t*_ is the time fixed effect, and ε_*it*_ is the error term for the individual family *i* in year *t*.

The existing literature presents various arguments, based on studies from different countries, about the negative correlation between health and income inequality (Omer et al., 2014; Validova, 2022). In the context of China, Li et al. (2015) explored the relationship between family assets and liabilities and happiness. They suggested health as a potential influencing factor, noting that greater financial liabilities can adversely affect an individual’s physical and psychological health. While research specifically focusing on the relationship between wealth inequality and health is limited, income inequality is generally believed to negatively impact health (Marmot, 2002).

On the other hand, marriage is often seen as positively contributing to happiness, offering emotional, social, economic, physical, and sexual support (Wadsworth, 2016). However, it has also been observed that disadvantaged groups, including those in poverty, face more challenges in getting married (Coontz and Folbre, 2002; Edin and Reed, 2005).

Given this backdrop, our review of the literature relating to wealth inequality and happiness, particularly in the realms of health and marriage, has led us to believe that both health and marriage could act as mediating factors. These aspects could play significant roles in elucidating the underlying mechanisms of the causal relationship between wealth inequality and happiness. Exploring these mediating effects can provide deeper insights into how wealth inequality influences happiness, particularly at the micro level.

#### Moderating effect model and variables

In our approach to analyzing the moderating effect, we have enhanced the original model (2) by introducing two additional variables. The first of these is the variable representing the moderating effect itself. The second variable is an interaction term, which was constructed by combining the wealth inequality variable and the moderating effect variable. The moderating effect model is presented below:

$$h\,a\,p\,p\,i\,n\,e\,s\,s_{i\,t}=\beta_{0}+\beta_{1}\,R\,D_{i\,t}+\beta_{2}\,m\,o\,d\,e\,r\,a\,t\,i\,n\,g_{i\,t}+$$

(5) $$\beta_{3}\,R\,D_{i\,t}*m\,o\,d\,e\,r\,a\,t\,i\,n\,g_{i\,t}+X_{i\,t}+\mu_{i}+\lambda_{t}+\varepsilon_{i\,t}$$

The *moderating*_*it*_ is the moderating effect variable for household *i* at year *t*. The *RD*_it_*moderating_*it*_ is the interactive variable between the moderating effect variable and wealth inequality. In our study, we considered social security expenditure in the percentage of government spending and the nominal GDP per capita of the province as two moderating variables. The *happiness*_*it*_ is the observed happiness for family *i* at year *t*, *RD*_*it*_ is the wealth inequality (wineq) index of an individual assigned from family wealth at year *t* based on KI, *X*_*it*_ is the control variables for family *i* in year *t*, μ_*i*_ is the province fixed effect, λ_*t*_ is the time fixed effect, and ε_*it*_ is the error term for the individual family *i* in year *t*.

In addition to mediating effects, we also considered the potential moderating effects of macroeconomic variables on the relationship between wealth inequality and happiness. For instance, government social security expenditures, particularly when targeted at addressing poverty and healthcare issues, are believed to positively influence happiness (Galama et al., 2017; Nordheim and Martinussen, 2020). Additionally, reforms in pension funds have been linked to increased happiness among beneficiaries (Pak, 2020).

Another aspect to consider is the role of local economic development in the correlation between wealth inequality and happiness. The literature presents mixed views on this topic. Easterlin et al. (2012) and Diener et al. (2013) have suggested that the relationship between absolute income growth and happiness may no longer hold in China and across a broad range of nations. Conversely, other studies, including Sacks et al. (2012), have posited a positive correlation between increases in absolute income and happiness, both in cross-country comparisons and within countries over time. More recent research in China, such as the study by Cai et al. (2023), has supported the notion that economic growth can indeed enhance happiness. These varying perspectives highlight the complexity of the interplay between economic factors and happiness, underscoring the need for our research to examine these potential moderating influences in the context of wealth inequality and happiness.

## Results and discussion

### Main result

In this part, we present our OLS regression result, as outlined in the following Table 4. There are four equations that we considered: equation (1) uses the panel OLS regression method to regress wineq directly against happiness, including only the time fixed effect; equation (2) uses the panel OLS regression method to regress wineq and other control variables together against happiness, including only the time fixed effect; equation (3) uses the panel OLS regression method to regress wineq directly against happiness, including both the time and province fixed effect; and equation (4) uses the panel OLS regression method to regress wineq and other control variables together against happiness, including both the time and province fixed effect.

**TABLE 4:** OLS regression result.

	(1)	(2)	(3)	(4)
	Happiness	Happiness	Happiness	Happiness
wineq	−0.2962***	−0.3192***	−0.3621***	−0.4162***
	(0.0405)	(0.0474)	(0.0482)	(0.0551)
male		−0.0053		0.0016
		(0.0209)		(0.0201)
age_60		0.2449***		0.2507***
		(0.0286)		(0.0284)
edu_year		−0.0106***		−0.0120***
		(0.0027)		(0.0026)
religion		0.0363		0.0378
		(0.0351)		(0.0327)
familysize		0.0035		0.0102*
		(0.0064)		(0.0059)
youth_rate		−0.0068		−0.0209
		(0.0750)		(0.0725)
elder_rate		−0.0224		−0.0230
		(0.0413)		(0.0421)
lfamily_income_n		0.0149		0.0169
		(0.0104)		(0.0103)
house		0.0718**		0.0419
		(0.0330)		(0.0334)
med		−0.0990***		−0.1001***
		(0.0342)		(0.0341)
_cons	3.4450***	3.2772***	3.1873***	3.0209***
	(0.0311)	(0.1301)	(0.0888)	(0.1524)
Time FE	Yes	Yes	Yes	Yes
Province FE			Yes	Yes
R^2^	0.2319	0.2377	0.2322	0.2378
*N*	13564	13564	13564	13564

Standard errors in parentheses.

*p < 0.1,

**p < 0.05

***p < 0.01.

Our initial hypothesis posits that an increase in wealth inequality (wineq) will lead to a decrease in subjective well-being. In examining this hypothesis, we first looked at equations (1) and (2), which include only the time fixed effect. In equation (1), the results show that high wineq is associated with decreased happiness (β = −0.2962, *p* < 0.01). When we introduced additional control variables in equation (2), the negative impact of wineq on happiness became even more pronounced (β = −0.3192, *p* < 0.01). This suggests that the control variables collectively contribute positively to happiness, enhancing the overall effect of wineq.

In equations (3) and (4), where we incorporated both time and provincial fixed effects, the relationship between relative wealth inequality and happiness was further accentuated. Specifically, the coefficient in equation (3) (β = −0.3621, *p* < 0.01) became more negative than in equation (1), and similarly, the coefficient in equation (4) (β = −0.4162, *p* < 0.01) became more negative than in equation (2). This indicates that when provincial differences are accounted for and households within the same province are compared, higher relative wealth inequality has a more pronounced negative effect on happiness. These findings collectively validate our first hypothesis, suggesting that increased wealth inequality is indeed correlated with a decrease in happiness.

Our analysis of control variables revealed several interesting findings related to happiness. Firstly, being over the age of 60 shows a significantly positive influence on happiness. This could be attributed to two factors: In China, reaching 60 typically signifies retirement and the commencement of pension fund benefits, which could contribute to increased happiness. Additionally, at this age, individuals might become less concerned with changing their social status and wealth, focusing instead on other life aspects, leading to greater contentment.

Another notable control variable is education. Our findings indicate that each additional year of education significantly decreases the happiness of a family head (*p* < 0.01). This could be explained by the higher expectations that often accompany higher education. Individuals with more education might anticipate higher incomes and better job prospects, leading to frustration and lower happiness when these expectations are not met, as suggested by Ruiu and Ruiu (2019). We will discuss the education variable further in the heterogeneity analysis section.

Lastly, catastrophic health payments have a negative effect on happiness. The reasons for this are two-fold: financially, bearing more than 40% of medical expenses out of pocket can be a considerable strain, potentially signaling a significant decline in life quality. Emotionally, catastrophic health payments often imply that a family member is suffering from a severe illness, which can be a source of distress for the entire family. These factors combined can considerably diminish overall happiness.

Using panel data, we observed significant variances in the beta coefficient, particularly when introducing certain control variables and the provincial fixed effect. This led us to propose several potential mechanisms:

Firstly, at the micro level, we hypothesize that there are key factors affecting the life quality of the family head that, in turn, influence their happiness. This feeling of happiness (or lack thereof) could then be transferred to other family members. Secondly, at the macro level, we consider the impact of regional policies that vary across provinces in China. For instance, the Chinese government’s efforts in poverty elimination have been substantial and successful in several regions. We posit that these policy initiatives, particularly those aimed at reducing wealth inequality, might contribute to lowering the wineq coefficient, thereby influencing household happiness.

The later parts of this research will delve into these proposed mechanisms in greater detail. We aim to explore how both micro and macro factors interact with wealth inequality to shape the happiness of households, offering a more nuanced understanding of the complex dynamics at play.

### Tests of consistency results

In this part of results, we have employed three different methods to test the consistency of our primary outcome. Firstly, we altered our regression approach to ordered probit regression. This change in methodology allowed us to examine whether our results hold under a different statistical framework. Secondly, we transformed the dependent variable from its original five-point scoring system to a binary variable format. This modification provides a different perspective on how happiness is quantified and interpreted in our analysis. Thirdly, we adjusted our independent variable, shifting from its nominal value representation to a real value basis. This recalibration aims to assess the impact of economic fluctuations and inflation on our findings.

#### Changing regression method

In this section, we present the results of our first Test of Consistency, as shown in Table 5. For this test, we switched from the OLS regression method to ordered probit regression. Ordered probit regression is often regarded as a more conventional method in happiness studies, primarily due to its ability to construct a non-linear relationship between variables such wealth inequality and happiness. Unlike OLS regression, which presumes a linear relationship, ordered probit regression allows for a more complex, non-linear interplay between the variables. Despite this methodological difference, Ferrer-i-Carbonell and Frijters (2004) have noted that in happiness research, OLS regression can yield results that are similar to those obtained from the ordered probit model. Therefore, we used panel ordered probit regression as our first method of consistency check, aiming to validate the robustness of our findings and ensure that our conclusions are not an artifact of the specific statistical approach employed.

**TABLE 5:** Test of Consistency 1: Changing Regression Method.

	(1)	(2)	(3)	(4)
	Happiness	Happiness	Happiness	Happiness
wineq	−0.3022***	−0.3455***	−0.3791***	−0.4612***
	(0.0438)	(0.0499)	(0.0489)	(0.0546)
male		−0.0056		0.0043
		(0.0210)		(0.0213)
age_60		0.2776***		0.2839***
		(0.0313)		(0.0315)
edu_year		−0.0143***		−0.0159***
		(0.0026)		(0.0026)
religion		0.0436		0.0449
		(0.0387)		(0.0389)
familysize		0.0031		0.0113*
		(0.0066)		(0.0067)
youth_rate		−0.0055		−0.0208
		(0.0831)		(0.0828)
elder_rate		−0.0014		−0.0014
		(0.0475)		(0.0474)
lfamily_income_n		0.0161		0.0181*
		(0.0104)		(0.0104)
house		0.0798**		0.0446
		(0.0348)		(0.0349)
med		−0.1116***		−0.1125***
		(0.0350)		(0.0349)
Time FE	Yes	Yes	Yes	Yes
Province FE			Yes	Yes
cut1				
_cons	−1.7852***	−1.6330***	−1.4990***	−1.3513***
	(0.0379)	(0.1250)	(0.1718)	(0.2087)
cut2				
_cons	−1.1322***	−0.9793***	−0.8455***	−0.6978***
	(0.0332)	(0.1238)	(0.1710)	(0.2082)
cut3				
_cons	0.1308***	0.2875**	0.4193**	0.5703***
	(0.0307)	(0.1233)	(0.1709)	(0.2082)
cut4				
_cons	0.9833***	1.1448***	1.2720***	1.4280***
	(0.0324)	(0.1236)	(0.1713)	(0.2085)
sigma2_u				
_cons	0.2325***	0.2207***	0.2100***	0.1965***
	(0.0207)	(0.0204)	(0.0201)	(0.0197)
chi2	1745.14	1900.16	1882.29	2047.33
*N*	13564	13564	13564	13564

Standard errors in parentheses.

*p < 0.1,

**p < 0.05,

***p < 0.01.

There are four equations we considered: equation (1) uses the panel ordered probit method to regress wineq directly against happiness, including only the time fixed effect; equation (2) uses the panel ordered probit method to regress wineq and other control variables together against happiness, including only the time fixed effect; equation (3) uses the panel ordered probit method to regress wineq directly against happiness, including both the time and province fixed effect; and equation (4) uses the panel ordered probit method to regress wineq and other control variables together against happiness, including both the time and province fixed effect.

In our Test of Consistency 1, we altered our measurement approach to utilize ordered probit regression. This change revealed that an increase in wealth inequality (wineq) consistently has a negative impact on happiness. This pattern held across different panels, whether we included control variables or not and regardless of whether we applied only a time fixed effect or both time and provincial fixed effects. While the specific coefficients obtained from this test differed from those in our original analysis, the direction and significance of these coefficients across all four equations remained consistent with the findings from the OLS model. This consistency in results, irrespective of the regression method used, allows us to confidently conclude that the outcomes of Test of Consistency 1 align with and support the conclusions drawn from our original OLS model analysis.

#### Dependent variable substitution

In this part, we present our Test of Consistency 2, as outlined in Table 6, in which we replaced the dependent variable from a five-point scale with a dummy variable. There are four equations we considered: equation (1) uses the panel OLS method to regress wineq directly against happiness, including only the time fixed effect; equation (2) uses the panel OLS method to regress wineq and other control variables together against happiness, including only the time fixed effect; equation (3) uses the panel OLS method to regress wineq directly against happiness, including both the time and province fixed effect; and equation (4) uses the panel OLS method to regress wineq and other control variables together against happiness, including both the time and province fixed effect.

**TABLE 6:** Test of Consistency 2: Dependent Variable Substitution.

	(1)	(2)	(3)	(4)
	Happiness_d	Happiness_d	Happiness_d	Happiness_d
wineq	−0.1301***	−0.1330***	−0.1518***	−0.1677***
	(0.0188)	(0.0224)	(0.0203)	(0.0240)
male		−0.0019		−0.0025
		(0.0097)		(0.0096)
age_60		0.0997***		0.1053***
		(0.0137)		(0.0134)
edu_year		−0.0037***		−0.0038***
		(0.0012)		(0.0011)
religion		0.0257		0.0264*
		(0.0167)		(0.0155)
familysize		0.0017		0.0045
		(0.0029)		(0.0028)
youth_rate		0.0100		0.0053
		(0.0320)		(0.0313)
elder_rate		−0.0136		−0.0161
		(0.0197)		(0.0199)
lfamily_income_n		0.0108**		0.0114**
		(0.0046)		(0.0045)
house		0.0117		0.0004
		(0.0157)		(0.0156)
med		−0.0159		−0.0175
		(0.0146)		(0.0145)
_cons	0.4581***	0.3498***	0.3933***	0.2728***
	(0.0148)	(0.0572)	(0.0415)	(0.0657)
Time FE	Yes	Yes	Yes	Yes
Province FE			Yes	Yes
R^2^	0.1996	0.2032	0.1996	0.2032
*N*	13564	13564	13564	13564

Standard errors in parentheses. *p < 0.1, **p < 0.05, ***p < 0.01.

In Test of Consistency 2, we modified our dependent variable, subjective well-being, to a binary format, assigning values of 0 and 1. Families with a happiness score of 3 or lower were labeled as ‘not happy’ (0), whereas those with scores above 3 were categorized as ‘happy’ (1). Utilizing this binary classification, we then applied the OLS model to re-assess all four equations, mirroring the approach used in our main result analysis. The results of this robustness test are detailed in Table 6.

The transformation of the dependent variable into a dummy format did not alter the direction and significance of the wealth inequality (wineq) coefficient. Moreover, the pattern of change observed in the coefficients across the four equations mirrored those noted in the five-point scale OLS results. Therefore, the findings from Test of Consistency 2 align with the outcomes derived from the five-point scale OLS regression, further affirming the reliability of our original results. This consistency reinforces the robustness of our conclusion that wealth inequality negatively impacts happiness.

#### Independent variable substitution

In this part, we presenting our Test of Consistency 3, as outlined in Table 7, in which we replace the independent variable from a nominal total asset to a comparable real total asset. There are four equations we considered: equation (1) uses the panel OLS method to regress wineq directly against happiness, including only the time fixed effect; equation (2) uses the panel OLS method to regress wineq and other control variables together against happiness, including only the time fixed effect; equation (3) uses the panel OLS method to regress wineq directly against happiness, including both the time and province fixed effect; and equation (4) uses the panel OLS method to regress wineq and other control variables together against happiness, including both the time and province fixed effect.

**TABLE 7:** Test of Consistency 3: Independent Variable Substitution.

	(1)	(2)	(3)	(4)
	Happiness	Happiness	Happiness	Happiness
wineq_adj	−0.2938***	−0.3382***	−0.3609***	−0.4362***
	(0.0410)	(0.0454)	(0.0483)	(0.0534)
male		−0.0045		0.0024
		(0.0212)		(0.0203)
age_60		0.2467***		0.2544***
		(0.0281)		(0.0280)
edu_year		−0.0104***		−0.0117***
		(0.0027)		(0.0026)
religion		0.0376		0.0403
		(0.0362)		(0.0337)
familysize		0.0004		0.0071
		(0.0065)		(0.0059)
youth_rate		−0.0082		−0.0218
		(0.0756)		(0.0725)
elder_rate		−0.0296		−0.0319
		(0.0412)		(0.0420)
lfamily_income_r		0.0027		0.0043
		(0.0053)		(0.0052)
house		0.0692**		0.0391
		(0.0325)		(0.0329)
med		−0.1189***		−0.1206***
		(0.0320)		(0.0317)
_cons	3.4390***	3.4071***	3.1835***	3.1598***
	(0.0308)	(0.0863)	(0.0888)	(0.1167)
Time FE	Yes	Yes	Yes	Yes
Province FE			Yes	Yes
R^2^	0.2336	0.2398	0.2339	0.2399
*N*	13564	13564	13564	13564

Standard errors in parentheses. **p < 0.05, ***p < 0.01.

In our third robustness test, we adjusted the total asset value of all families using the consumer price index (CPI) data published by the World Bank, with the base year set at 2010 (2010 = 100). Alongside this, we also recalibrated the average family member income among the control variables for comparability. Following these adjustments, we recalculated the Kakwani Index (KI) to represent the level of wealth inequality more accurately in real terms.

The results of Test of Consistency 3, as shown in Table 7, indicate that while the coefficients across all four equations became more negative, their direction and significance remained unchanged. This outcome underscores the robustness of our original findings, demonstrating that the use of nominal values for assets and other control variables does not significantly alter the statistical results. This consistency in findings, regardless of the adjustment for inflation, reinforces the validity of our conclusions about the relationship between wealth inequality and subjective well-being.

In robustness test 3, we adjusted the total asset value for all families using consumer price index data published by the World Bank using 2010 = 100, and we also adjusted the average family member income in control variables to make it comparable. After adjustment, we recalculated the KI and used it as a representation of the wealth inequality level. The Test of Consistency 3 regression is shown in Table 7. The coefficient for equations (1) and (3) became less negative, while equations (2) and (4) became more negative, but the direction and significance remained the same. This consistency shows the use of nominal value for assets and other control variables did not statistically influence our results.

#### Endogeneity test

In this part, we wanted to check the potential endogeneity issue within the OLS model. We proposed two methods. In equations (1) and (2), we used daily necessity expenditure (daily_exp) and electric expenditure (electric_exp) as IVs for the 2SLS test. In equation (3), we used the time and family fixed effects instead of time and province fixed effects to test endogeneity. Equation (4) is the original OLS equation. The results are presented in Table 8.

**TABLE 8:** Endogeneity Test.

	1st stage	2nd stage	Family FE	OLS
	(1)	(2)	(3)	(4)
	wineq	happiness	happiness	happiness
wineq			−0.1820**	−0.4171***
			(0.0843)	(0.0547)
wineq_iv		−0.7120*		
		(0.3984)		
daily_exp	−0.0002***			
	(0.0000)			
electric_exp	−0.0001***			
	(0.0000)			
male	0.0155***	0.0036	0.0018	−0.0003
	(0.0038)	(0.0215)	(0.0317)	(0.0199)
age_60	−0.0012	0.2529***	0.2439***	0.2519***
	(0.0050)	(0.0282)	(0.0441)	(0.0279)
edu_year	−0.0090***	−0.0136***	−0.0128***	−0.0110***
	(0.0006)	(0.0044)	(0.0049)	(0.0026)
religion	−0.0126	0.0309	0.0347	0.0342
	(0.0078)	(0.0327)	(0.0490)	(0.0318)
familysize	−0.0141***	0.0046	0.0052	0.0093
	(0.0015)	(0.0090)	(0.0119)	(0.0060)
youth_rate	−0.0101	−0.0175	0.0321	−0.0141
	(0.0135)	(0.0733)	(0.1234)	(0.0735)
elder_rate	−0.0082	−0.0247	−0.0655	−0.0224
	(0.0072)	(0.0425)	(0.0495)	(0.0424)
lfamily_income_n	−0.0476***	0.0026	−0.0044	0.0155
	(0.0035)	(0.0218)	(0.0125)	(0.0102)
house	−0.0849***	0.0212	0.0123	0.0459
	(0.0100)	(0.0458)	(0.0431)	(0.0327)
med	0.0095	−0.0972***	−0.0859**	−0.1001***
	(0.0065)	(0.0341)	(0.0434)	(0.0335)
_cons	0.9996***	3.3058***	3.4171***	3.0265***
	(0.0436)	(0.4056)	(0.1701)	(0.1530)
Time FE	Yes	Yes	Yes	Yes
Province FE	Yes	Yes		Yes
Family FE			Yes	
R^2^	0.1246	0.2369	0.2379	0.2373
*N*	13564	13564	13564	13564

Standard errors in parentheses. *p < 0.1, **p < 0.05, ***p < 0.01.

In equation (1), we inferred that consumption of daily necessities and electricity expenditure are inversely related to wealth inequality. The rationale for selecting these two independent variables (IVs) is twofold: firstly, daily necessity consumption tends to vary with wealth as wealthier individuals often opt for higher quality products, resulting in increased spending on these necessities. Secondly, electricity expenditure is associated with wealth, particularly in Chinese families, where the house constitutes a significant portion of wealth. This expenditure tends to be proportional to the size of the house. These IVs, according to the “set point theory,” do not have a direct correlation with happiness once basic life necessities are met. Therefore, we considered daily necessity and electricity expenditures to be suitable IVs for this analysis.

In equation (2), we employed the first-stage coefficients to compute the wineq_iv and subsequently regress it against happiness. The resulting coefficient was −0.7120, with a significance level of *p* < 0.10. This coefficient, though less significant than the OLS coefficient (β = −0.4171, *p* < 0.01), maintained the same direction. In equation (3), we replaced province-level FE with family-level FE to address potential OVB. This adjustment resulted in the wineq coefficient retaining its direction but exhibiting reduced significance (β = −0.1820, *p* < 0.05). A possible explanation for this diminished significance is that incorporating family-level FE reduced variance within the dataset. Overall, the application of IV and lower-level fixed effects validates that the conclusions drawn from our OLS method are robust and not compromised by endogeneity issues.

### Heterogeneity analysis results

In this part of the results and discussions, we have focused on three heterogeneity issues. Our first area of focus was the difference attributable to possessing rural versus urban hukou. In addition, our analysis also considered the heterogeneity associated with education levels and family member incomes. We believe that these factors could yield insightful variations in the relationship between wealth inequality and happiness.

#### Hukou

Hukou, a household registration system in China, designates individuals as permanent residents of a specific area and influences access to various benefits, including education, healthcare, and retirement pensions. The type of hukou one holds can thus significantly affect their lifestyle and opportunities throughout their life. Based on existing literature, such as Jiang et al. (2008), Tani (2017), and Lu and Wu (2020), we acknowledge the potential for heterogeneity in our findings. Thus, we intended to reexamine how changes in wealth inequality impact happiness among different hukou categories, using a fixed effects model to account for these variations.

We summarize the Hukou heterogeneity table in Table 9. Equation (1) and (2) are the regression of rural hukou holders; equation (3) and (4) are the regression of urban hukou holders.

**TABLE 9:** Heterogeneity Regression Results of Hukou.

Hukou category	Rural Hukou		Urban Hukou	
	(1)	(2)	(3)	(4)
	Happiness	Happiness	Happiness	Happiness
wineq	−0.4566***	−0.5228***	−0.0868	−0.1865**
	(0.0653)	(0.0697)	(0.0754)	(0.0899)
male	−0.0213	−0.0110	0.0151	0.0193
	(0.0234)	(0.0223)	(0.0400)	(0.0397)
age_60	0.2165***	0.2216***	0.3193***	0.3078***
	(0.0355)	(0.0346)	(0.0561)	(0.0542)
edu_year	−0.0081***	−0.0094***	−0.0122**	−0.0155***
	(0.0031)	(0.0028)	(0.0054)	(0.0053)
religion	0.0188	0.0206	0.0860	0.0861
	(0.0437)	(0.0408)	(0.0587)	(0.0596)
familysize	−0.0045	0.0039	0.0306**	0.0344***
	(0.0077)	(0.0069)	(0.0120)	(0.0119)
youth_rate	0.0200	0.0111	−0.0185	−0.0363
	(0.0859)	(0.0830)	(0.1636)	(0.1625)
elder_rate	0.0344	0.0238	−0.1360**	−0.1243*
	(0.0515)	(0.0522)	(0.0671)	(0.0663)
lfamily_income_n	−0.0017	−0.0041	0.1105***	0.1157***
	(0.0106)	(0.0105)	(0.0233)	(0.0253)
house	0.0169	−0.0142	0.1544***	0.1264**
	(0.0409)	(0.0405)	(0.0490)	(0.0528)
med	−0.1065***	−0.1116***	−0.0682	−0.0600
	(0.0383)	(0.0383)	(0.0853)	(0.0856)
_cons	3.5848***	3.0295***	2.1058***	1.9376***
	(0.1440)	(0.3921)	(0.2578)	(0.2995)
Time FE	Yes	Yes	Yes	Yes
Province FE		Yes		Yes
R^2^	0.2449	0.2460	0.2272	0.2262
*N*	10100	10100	3464	3464

Standard errors in parentheses. *p < 0.1, **p < 0.05, ***p < 0.01.

Our analysis, as presented in Table 9, revealed a notable difference in how wealth inequality affects the happiness of rural and urban hukou holders in China. Comparing the wineq coefficients from equations (1) and (3), we observed distinct patterns:

In equation (1), the coefficient for rural hukou holders is −0.4566, significant at the 1% level. Conversely, in equation (3), for urban hukou holders, the coefficient is −0.0868, lacking statistical significance. This suggests that without considering provincial fixed effects, urban hukou holders appear less sensitive to wealth inequality, while rural hukou holders experience a more significant negative impact. However, when provincial fixed effects are included, as in equations (2) and (4), both coefficients become more negative and statistically significant. For rural hukou holders (equation 2), the coefficient deepens to −0.5228 (*p* < 0.01), and for urban hukou holders (equation 4), it reaches −0.1865 (*p* < 0.05). This indicates that, upon controlling for provincial variances, wealth inequality adversely affects urban hukou holders as well, albeit less severely than their rural counterparts. These findings align with Zhao (2017), who noted that transitioning from rural to urban hukou status could enhance individual happiness. They differ from earlier research, such as that of Knight et al. (2009), who suggested that rural residents might experience higher subjective happiness due to limited information access and a narrower perspective.

We hypothesize that the advantages associated with urban hukou status, such as better healthcare access and overall health conditions, could be contributing factors. Furthermore, literature such as that of Hu (2016) highlights the significant role of hukou status in aspects of social life, such as marriage. These elements could be potential mechanisms behind the varying impacts of wealth inequality on happiness among rural and urban hukou holders.

#### Level of education

Next, we analyzed the level of education heterogeneity, and we have summarized the results in the Table 10. Equation (1) is for people who received no education; equation (2) is for people who finished primary school education; equation (3) is for people who finished middle school education; and equation (4) is for people who finished high school or higher education.

**TABLE 10:** Heterogeneity Regression Results of Level of Education.

Level of education	Illiterate or semi-illiterate edu_year = 0	Primary School edu_year = 6	Middle School edu_year = 9	High School or above edu_year > = 12
	(1)	(2)	(3)	(4)
	Happiness	Happiness	Happiness	Happiness
wineq	−0.6348***	−0.5437***	−0.3057***	−0.2350**
	(0.1270)	(0.1027)	(0.0769)	(0.1054)
male	−0.0400	−0.0238	0.0376	0.0277
	(0.0422)	(0.0355)	(0.0324)	(0.0449)
age_60	0.2792***	0.2836***	0.2277***	0.2883***
	(0.0480)	(0.0560)	(0.0574)	(0.0687)
religion	0.0751	−0.0580	0.0864	0.0482
	(0.0636)	(0.0656)	(0.0606)	(0.0869)
familysize	−0.0135	0.0028	0.0211**	0.0465***
	(0.0122)	(0.0105)	(0.0099)	(0.0145)
youth_rate	0.0877	0.1180	−0.0322	−0.2306
	(0.1403)	(0.1470)	(0.1368)	(0.1596)
elder_rate	−0.0190	−0.1112	−0.1196	−0.0308
	(0.0750)	(0.0781)	(0.0840)	(0.0827)
lfamily_income_n	0.0072	0.0192	0.0326**	0.0370
	(0.0192)	(0.0143)	(0.0166)	(0.0280)
house	−0.0274	−0.0921	0.1716***	0.0956*
	(0.0641)	(0.0714)	(0.0610)	(0.0561)
med	−0.1519***	−0.0298	−0.0574	−0.1526
	(0.0568)	(0.0583)	(0.0631)	(0.0954)
_cons	4.5564***	3.7608***	2.4820***	2.3428***
	(0.2412)	(0.2975)	(0.2264)	(0.4258)
Time FE	Yes	Yes	Yes	Yes
Province FE	Yes	Yes	Yes	Yes
R^2^	0.2886	0.2374	0.2223	0.2228
*N*	3,734	3,464	4,011	2,355

Standard errors in parentheses. *p < 0.1, **p < 0.05, ***p < 0.01.

The analysis presented in Table 10 indicates that individuals with higher levels of education exhibit less sensitivity to wealth inequality in terms of their happiness. This observation is evidenced by the decreasing wineq coefficients as the level of education increases. Interestingly, this finding appears to be at odds with our main result, which suggests that an increase in years of education leads to decreased happiness, assuming wealth inequality remains constant. These seemingly contradictory findings may actually represent the dual influences of education on happiness. The main result reflects the negative aspect of education on happiness. It suggests that education raises both job outcomes and expectations, leading to increased frustration when actual job outcomes do not meet these heightened expectations (Ruiu and Ruiu, 2019). In contrast, our heterogeneity analysis, which categorizes groups based on different education levels, demonstrates that education reduces the impact of wealth inequality on happiness.

A possible explanation for this reduced sensitivity is that higher education might enable individuals to perceive their lives through a broader lens that extends beyond mere wealth accumulation (Nikolaev, 2018). In essence, while education may increase expectations and potential for frustration in some areas, it also appears to provide individuals with a more resilient perspective that buffers the negative effects of wealth inequality on their happiness.

#### Average family member income

Finally, our study delved into the heterogeneity related to varying levels of family member incomes. We utilized the CFPS data, which offer a comparable measure of average family member income derived from the family economic survey section. This specific data element allowed us to conduct a comparative analysis of family member incomes between 2012 and 2018, adjusted to the 2010 price level. We cut the family member’s average income to four quartiles. Equation (1) captures the sensitivity of happiness against wealth inequality for families with the lowest 25% of the average family income; equation (2) captures the 25–50% quartile; equation (3) captures the 50–75% quartile; and equation (4) captures the top 25% quartile. The regression result is presented in Table 11.

**TABLE 11:** Heterogeneity Regression Results of Average Family Member Income.

Income quartile	1st quartile	2nd quartile	3rd quartile	4th quartile
	(1)	(2)	(3)	(4)
	Happiness	Happiness	Happiness	Happiness
wineq	−0.7371***	−0.4335***	−0.3692***	−0.2536***
	(0.1048)	(0.1121)	(0.1002)	(0.0855)
male	−0.0254	0.0122	0.0022	0.0005
	(0.0445)	(0.0345)	(0.0387)	(0.0325)
age_60	0.3065***	0.2506***	0.2721***	0.1747***
	(0.0542)	(0.0579)	(0.0556)	(0.0499)
edu_year	−0.0109**	−0.0087*	−0.0073	−0.0226***
	(0.0049)	(0.0047)	(0.0049)	(0.0045)
religion	0.1150*	−0.0232	0.0019	0.0469
	(0.0653)	(0.0668)	(0.0612)	(0.0726)
familysize	0.0159	−0.0054	0.0218**	0.0116
	(0.0122)	(0.0117)	(0.0108)	(0.0115)
youth_rate	−0.0041	−0.1119	0.1925	−0.0460
	(0.1286)	(0.1466)	(0.1395)	(0.1900)
elder_rate	−0.0136	−0.0975	0.0047	0.0330
	(0.0906)	(0.0886)	(0.0914)	(0.0714)
lfamily_income_n	−0.0062	−0.0046	−0.0061	0.0461
	(0.0140)	(0.0418)	(0.0489)	(0.0365)
house	0.0313	−0.0735	0.0942	0.1030*
	(0.0695)	(0.0744)	(0.0645)	(0.0553)
med	−0.0699	−0.1289*	−0.1036	−0.1634*
	(0.0459)	(0.0711)	(0.1006)	(0.0938)
_cons	4.7985***	3.2255***	2.9705***	2.8806***
	(0.1921)	(1.2120)	(0.5078)	(0.4005)
Time FE	Yes	Yes	Yes	Yes
Province FE	Yes	Yes	Yes	Yes
R^2^	0.2623	0.2417	0.2594	0.2250
*N*	3392	3390	3391	3391

Standard errors in parentheses. *p < 0.1, **p < 0.05, ***p < 0.01.

Table 11 reveals that the coefficient for wealth inequality (wineq) in equation (1) is most negative, indicating the lowest 25% income families’ happiness is most sensitive to wealth inequality. As the average income per family member increases, the negative impact of wineq on happiness diminishes. This observation aligns with Khalil’s (2019) notion that happiness stems from the production of corporal utility within a given context, often achieved through consumption. Wealth, as defined by Clark et al. (2008), represents an accumulation of income and a means to extend past consumption power into the future. Therefore, for families with higher average incomes, the reliance on accumulated wealth for happiness lessens. They can more readily satisfy their consumption needs through current income, which, in turn, generates utility and contributes to happiness. As a result, the influence of wealth inequality on their happiness decreases with increasing family income.

## Mechanism discussion

We have summarized both mediating effects’ results in Table 12 and both moderating effects’ results in Table 13.

**TABLE 12:** Mediating Effect Regression Results.

	(1)	(2)	(3)	(4)
	Health	Happiness	Marriage	Happiness
wineq	0.3542***	−0.3618***	−0.0350***	−0.4070***
	(0.0583)	(0.0533)	(0.0136)	(0.0545)
health		−0.1553***		
		(0.0082)		
marriage				0.1511***
				(0.0285)
male	−0.2429***	−0.0369*	0.0111*	−0.0006
	(0.0219)	(0.0199)	(0.0059)	(0.0201)
age_60	0.3435***	0.3060***	−0.0476***	0.2592***
	(0.0297)	(0.0284)	(0.0100)	(0.0284)
edu_year	−0.0173***	−0.0144***	0.0015*	−0.0122***
	(0.0028)	(0.0025)	(0.0009)	(0.0025)
religion	0.0076	0.0394	−0.0159	0.0424
	(0.0405)	(0.0313)	(0.0102)	(0.0328)
familysize	−0.0221***	0.0068	0.0387***	0.0041
	(0.0072)	(0.0058)	(0.0025)	(0.0058)
youth_rate	−0.6305***	−0.1262*	0.0165	−0.0247
	(0.0794)	(0.0705)	(0.0186)	(0.0729)
elder_rate	−0.1386***	−0.0426	−0.0507***	−0.0166
	(0.0497)	(0.0411)	(0.0130)	(0.0420)
lfamily_income_n	0.0181	0.0198**	0.0054*	0.0161
	(0.0122)	(0.0100)	(0.0030)	(0.0103)
house	−0.0175	0.0406	0.0245**	0.0371
	(0.0338)	(0.0330)	(0.0114)	(0.0334)
med	0.4497***	−0.0267	0.0163*	−0.1019***
	(0.0363)	(0.0336)	(0.0096)	(0.0339)
_cons	3.1659***	3.5108***	0.6479***	2.9242***
	(0.1616)	(0.1563)	(0.0482)	(0.1526)
Time FE	Yes	Yes	Yes	Yes
Province FE	Yes	Yes	Yes	Yes
R^2^	0.2886	0.2374	0.2223	0.2238
*N*	13564	13564	13564	13564

Standard errors in parentheses. *p < 0.1, **p < 0.05, ***p < 0.01.

**TABLE 13:** Moderating Effect Regression Table.

	(1)	(2)	(3)	(4)
	Happiness	Happiness	Happiness	Happiness
wineq × social_security_exp	0.6532*	0.7997**		
	(0.3335)	(0.3225)		
social_security_exp	1.3569***	1.8808**		
	(0.3801)	(0.8338)		
wineq × ngdppc			0.0259**	0.0237**
			(0.0108)	(0.0102)
ngdppc			−0.3193***	−0.3131
			(0.1099)	(0.5284)
wineq	−0.3742***	−0.4314***	−0.4007***	−0.4116***
	(0.0509)	(0.0551)	(0.0580)	(0.0551)
male	0.0057	0.0025	−0.0106	0.0014
	(0.0208)	(0.0202)	(0.0211)	(0.0201)
age_60	0.2477***	0.2523***	0.2597***	0.2511***
	(0.0287)	(0.0282)	(0.0288)	(0.0284)
edu_year	−0.0119***	−0.0120***	−0.0102***	−0.0117***
	(0.0026)	(0.0025)	(0.0027)	(0.0026)
religion	0.0349	0.0323	0.0357	0.0362
	(0.0345)	(0.0330)	(0.0348)	(0.0330)
familysize	0.0067	0.0098*	0.0004	0.0101*
	(0.0063)	(0.0059)	(0.0066)	(0.0059)
youth_rate	−0.0054	−0.0317	−0.0232	−0.0229
	(0.0744)	(0.0711)	(0.0740)	(0.0707)
elder_rate	−0.0340	−0.0340	−0.0241	−0.0272
	(0.0413)	(0.0413)	(0.0412)	(0.0420)
lfamily_income_n	0.0137	0.0166	0.0187*	0.0170
	(0.0101)	(0.0102)	(0.0104)	(0.0104)
house	0.0518	0.0238	0.0369	0.0251
	(0.0345)	(0.0335)	(0.0344)	(0.0335)
med	−0.0957***	−0.0995***	−0.0949***	−0.0998***
	(0.0339)	(0.0340)	(0.0342)	(0.0340)
_cons	3.1588***	2.8909***	4.7805***	4.6210*
	(0.1335)	(0.1751)	(0.5339)	(2.6111)
Time FE	Yes	Yes	Yes	Yes
Province FE		Yes		Yes
R^2^	0.2395	0.2399	0.2374	0.2381
*N*	13564	13564	13564	13564

Standard errors in parentheses. *p < 0.1, **p < 0.05, ***p < 0.01.

### Mediating effect of health

In Table 12, equation (1) initially focuses on the regression of health against wealth inequality. Subsequently, equation (2) extends this analysis to include both health and wealth inequality as variables influencing happiness. The integration of these two equations substantiates the role of health as a mediator in the causal link between wealth inequality and happiness. Specifically, equation (1) reveals that an increase in wealth inequality (wineq) is associated with deteriorated health outcomes. In the CFPS data, a higher health score indicates poorer health, suggesting that greater deprivation leads to poorer health. Furthermore, equation (2) demonstrates a negative, statistically significant impact of health on happiness, indicating that declining health leads to reduced happiness.

This observation aligns with existing research, such as that of Siahpush et al. (2008) and Sabatini (2014), which has found a positive relationship between health and happiness. Concurrently, our findings are consistent with Li et al. (2015), showing a negative link between relative wealth and health. Therefore, health is considered a mediating factor that can intensify the impact of wealth inequality on happiness.

### Mediating effect of marriage

In Table 12, equation (3) initially examines the relationship between marriage and wealth inequality, followed by an analysis in equation (4) that considers both marriage and wealth inequality in relation to happiness. The outcomes of these equations suggest that marriage acts as a mediator in the relationship between wealth inequality and happiness. Specifically, equation (3) reveals a negative and significant relationship between wealth inequality (wineq) and marriage at the 1% level. This aligns with the findings of Coontz and Folbre (2002) and Edin and Reed (2005), who observed that individuals in more deprived circumstances are less likely to marry.

Furthermore, equation (4) identifies a positive effect of marriage on happiness, corroborating the extensive literature in this domain. By integrating these two coefficients, it becomes evident that deprivation not only reduces the likelihood of marriage but also negatively impacts happiness through this marital disadvantage. Consequently, marriage is recognized as another mediating variable that amplifies the adverse effects of wealth inequality on happiness.

#### Moderating effect of social security expenditure

Next, our study explored the influence of government social security expenditure on the relationship between wealth inequality and happiness by employing a moderating effect analysis. This involved incorporating both the percentage of social security spending in provincial government total expenditure and an interactive variable that combines social security spending percentage with wineq.

As outlined in Table 13, equations (1) and (2) were used to estimate this moderating effect on happiness. Equation (1) accounts for time fixed effects, while equation (2) includes both time and province fixed effects. The findings from equation (1) indicate that social security spending has a positive and significant impact on happiness. Additionally, the interactive variable between social security spending and wineq, despite being significant only at the 10% level, also exhibits a positive effect.

In equation (2), with the inclusion of province fixed effects, the social security expenditure coefficient, despite lowering the significance to 5%, remains positive. As for the interactive variable, it exhibits increased significance from 10 to 5%. This variation indicates that the effects of social security spending may differ across provinces. Notably, regardless of these provincial differences, social security expenditure appears to most benefit those experiencing higher levels of wealth inequality, such as those feeling greater inequity or poverty. The increase in government social security expenditure seems to mitigate the impact of wealth inequality on happiness, primarily through its role in reducing poverty. This finding aligns with existing literature on the subject.

#### Moderating effect of economic development

In this section, the analysis included the province-level nominal GDP per capita and its interactive variable with wineq. Using equations (3) and (4) from Table 13, the study assessed the impact of nominal GDP per capita on happiness and how the interaction between GDP per capita and wineq influences happiness. Equation (3), which incorporates only the time fixed effect, reveals that GDP per capita negatively correlates with happiness, and this correlation is statistically significant at the 1% level.

This analysis revealed that the interactive variable, representing the relationship between GDP per capita and wealth inequality, is positively correlated with happiness and is significant at the 5% level. This suggests that individuals residing in provinces with higher economic development and greater wealth inequality tend to be happier than those living in provinces with lower economic development. The proposed explanation for this phenomenon is the demonstration effect, where people maintain a positive outlook on their future, believing that they can achieve a status similar to their more affluent peers. This optimism persists even among those currently experiencing greater wealth inequality as they anticipate climbing the social ladder.

In equation (4) of the study, after accounting for the province fixed effect, the significance of the GDP per capita coefficient diminishes, while the interactive variable continues to show positive significance at the 5% level. This shift in significance is attributed to the relatively smaller differences in nominal GDP per capita within provinces compared to across the country. However, even as a province undergoes economic growth, those in more deprived situations still harbor a more optimistic view of the future, which contributes to their increased happiness.

This outcome challenges the findings of earlier research, such as that of Easterlin et al. (2012), but aligns with more recent studies, such as that of Cai et al. (2023). Cai et al. argue from the perspective of absolute utility, suggesting that in a developing country like China, the satisfaction of basic needs through increased absolute income enhances people’s happiness. Additionally, they observe that while the positive impact of economic growth on happiness typically lasts only a few years, China’s sustained high-speed economic growth extends this effect. This perspective is in line with the set point theory, which posits that happiness remains stable after basic life necessities are met but can vary in response to shocks in life. The continuous economic growth in China acts as a persistent positive shock on happiness, demonstrating a moderating effect that helps to explain the findings in the study.

## Conclusion and discussion

In our research, presented in this article, we examined the impact of relative wealth inequality on happiness, drawing on data from the CFPS. Our study has yielded several key findings. Firstly, we established that wealth inequality significantly and negatively affects happiness, a conclusion that remains consistent across various tests for robustness. Secondly, acknowledging the heterogeneity in our data as identified in existing research, we delved into various aspects: we explored the differential impact of wealth inequality on happiness between rural and urban hukou holders. Our analysis revealed that families with rural hukou experience a more pronounced negative reaction to wealth inequality compared to urban hukou holders. We also investigated how the education level of the family head influences the relationship between wealth inequality and happiness. Our findings suggest that family heads with higher education levels are less sensitive to the effects of wealth inequality. At last, in examining the impact of average member income within families, we discovered that families with higher per-member income are less adversely affected by wealth inequality. Upon completing the analysis of heterogeneity, we explored potential mechanisms driving these results. At the micro level, factors such as the health and marital status of the family head were found to intensify the feelings of unhappiness among those experiencing relative deprivation. Conversely, at the macro level, variables including the social security spending of provincial governments and the economic growth within provinces were observed to statistically mitigate the negative effects of relative wealth inequality on happiness.

Following our investigation into the mechanisms linking relative wealth inequality and happiness, we have proposed a set of policy recommendations aimed at enhancing happiness within society: First, enhancing the health focus. The government should prioritize the health of its citizens. This could involve initiatives to bolster health education and the provision of free or low-cost health examinations, especially for those experiencing greater wealth inequality. Second, promoting marriage incentives. To address the observed impact of marital status on happiness, the government could consider implementing incentives to encourage marriage. This could include tax exemptions or other economic benefits designed to increase the marriage rate, thereby alleviating wealth inequality and improving happiness through the channel of marriage. Third, increasing social security expenditure. Provincial governments should consider boosting their social security spending. However, it is crucial that these funds are allocated effectively and judiciously to ensure they are mitigating wealth inequality. Fourth, fostering economic development. Provincial governments should strive to promote economic development actively. As our study suggests, economic growth, particularly at the provincial level, can have a mitigating effect on the negative impact of wealth inequality on happiness and potentially lower wealth inequality.

Future studies may explore further in this field by considering the following directions. Firstly, our study relies on self-reported measures of happiness and wealth, which are inherently subjective. Future studies might benefit from incorporating more objective data, if available, to enhance the reliability of the findings. Secondly, while our analysis accounts for hukou heterogeneity, it does not consider changes in hukou status, such as the migration of individuals from rural to urban areas. This oversight could lead to unaddressed heterogeneity issues within this particular demographic. Thirdly, our study is constrained by the limitations of the available data, preventing a comprehensive exploration of more potential heterogeneity factors among families. An aspect worth investigating is the ownership of financial assets. The access to the securities market may influence individuals’ perceptions of happiness, creating a distinction between those who have such access and those who do not.

Our findings aim to contribute to the broader understanding of wealth inequality and happiness. The limitations and challenges we have identified, along with the mechanisms we have estimated, should serve as valuable directions for future studies in this field. By addressing these issues, subsequent research can build upon our work to develop a more nuanced understanding of the complex interplay between wealth inequality and happiness.

## Data availability statement

Publicly available datasets were analyzed in this study. This data can be found here: https://www.isss.pku.edu.cn/cfps/en/index.htm.

## Author contributions

JW: Writing—original draft, Writing—review and editing.
